# Tau Regulates Glioblastoma Progression, 3D Cell Organization, Growth and Migration via the PI3K-AKT Axis

**DOI:** 10.3390/cancers13225818

**Published:** 2021-11-19

**Authors:** Alessandra Pagano, Gilles Breuzard, Fabrice Parat, Aurélie Tchoghandjian, Dominique Figarella-Branger, Tiphany Coralie De Bessa, Françoise Garrouste, Alexis Douence, Pascale Barbier, Hervé Kovacic

**Affiliations:** 1 Faculté des Sciences Médicales et Paramédicales, Institut de Neurophysiopathologie (INP), Team 9, UMR 7051, CNRS, Aix Marseille Université, 13005 Marseille, France; gilles.breuzard@univ-amu.fr (G.B.); fabrice.parat@univ-amu.fr (F.P.); garroustefrancoise@gmail.com (F.G.); alexisdouence@gmail.com (A.D.); pascale.barbier@univ-amu.fr (P.B.); herve.kovacic@univ-amu.fr (H.K.); 2 Faculté des Sciences Médicales et Paramédicales, Institut de Neurophysiopathologie (INP), Team 8, UMR 7051, CNRS, Aix Marseille Université, 13005 Marseille, France; aurelie.tchoghandjian@univ-amu.fr (A.T.); dominique.figarella-branger@univ-amu.fr (D.F.-B.); 3 Service d’Anatomie Pathologique et de Neuropathologie, CHU Timone, APHM, 13005 Marseille, France; 4 LIM 64: Instituto do Coracao (InCor), Hospital das Clinicas HCFMUSP, Faculdade de Medicina, Universidade de Sao Paulo, Sao Paulo 05403-090, SP, Brazil; debessa@hotmail.fr

**Keywords:** Microtubule-Associated Protein Tau (MAPT), glioblastoma, N-cadherin, PI3 kinase (PI3K), Akt, multicellular spheroid

## Abstract

**Simple Summary:**

The Microtubule-associated protein Tau is expressed in different cancers; however, its role and prognostic value are still debated. In the present work, we evaluated the role of Tau in glioblastoma by down-regulating its expression in glioblastoma cells. We showed that Tau: (1) is required for tumor progression in nude mice; (2) is necessary for glioblastoma 3D cell organization, growth, and migration; and (3) regulates the PI3K/AKT signaling pathway.

**Abstract:**

The Microtubule-Associated Protein Tau is expressed in several cancers, including low-grade gliomas and glioblastomas. We have previously shown that Tau is crucial for the 2D motility of several glioblastoma cell lines, including U87-MG cells. Using an RNA interference (shRNA), we tested if Tau contributed to glioblastoma in vivo tumorigenicity and analyzed its function in a 3D model of multicellular spheroids (MCS). Tau depletion significantly increased median mouse survival in an orthotopic glioblastoma xenograft model. This was accompanied by the inhibition of MCS growth and cell evasion, as well as decreased MCS compactness, implying N-cadherin mislocalization. Intracellular Signaling Array analysis revealed a defective activation of the PI3K/AKT pathway in Tau-depleted cells. Such a defect in PI3K/AKT signaling was responsible for reduced MCS growth and cell evasion, as demonstrated by the inhibition of the pathway in control MCS using LY294002 or Perifosine, which did not significantly affect Tau-depleted MCS. Finally, analysis of the glioblastoma TCGA dataset showed a positive correlation between the amount of phosphorylated Akt-Ser473 and the expression of *MAPT* RNA encoding Tau, underlining the relevance of our findings in glioblastoma disease. We suggest a role for Tau in glioblastoma by controlling 3D cell organization and functions via the PI3K/AKT signaling axis.

## 1. Introduction

Glioblastomas (grade IV of gliomas, GBM) are the most frequent primary tumors of the central nervous system. They are very aggressive, highly angiogenic, and associated with a very bad prognosis. They account for 52% to 75% of diagnosed gliomas with a median patient survival of 15 months [1,2]. Glioblastomas are characterized by extensive genetic alterations, including amplification of the gene encoding the epidermal growth factor receptor (EGFR) and inactivating the mutation or deletion of Phosphatase and Tensin homolog deleted on chromosome 10 (PTEN), both promoting invasive behavior [3,4]. The invasive and angiogenic phenotype of GBM tumors renders surgical tumor resection difficult and limits the efficacy of current radiotherapy and chemotherapy. Therefore, the search for new targets and therapeutic approaches is still a current challenge.

Microtubules (MTs) and their dynamic instabilities are major players in cancer cell proliferation or invasiveness and the main targets of cancer therapy [5,6,7]. The Microtubule-associated Protein Tau (encoded by *MAPT*) stabilizes MTs and promotes their assembly [8,9,10]. *MAPT* gene transcription and Tau protein expression are altered in several cancers, such as breast, prostate, ovarian cancer and gliomas [11,12,13,14]. High levels of Tau expression have been associated with resistance to MTs-targeting agents, such as taxanes, in breast and ovarian cancer [11,12,13]. Therefore, Tau is considered as a predictive marker for the response to anti-mitotic-based therapy. In contrast, the role of Tau as a cancer prognostic marker is controverted. It has been shown that the risk of developing cancer is significantly higher in families affected by genetic tauopathies proposing that Tau may be a risk factor for cancer [14]. A correlation between Tau expression and poor disease outcome has been proposed in ovarian, prostate and colorectal cancer [15,16,17]. On the other hand, high levels of Tau expression have been positively correlated with better patient survival, independently of the therapy in breast cancer and low-grade gliomas (LGG) [18,19]. However, the exact role of Tau in high-grade gliomas (GBM) is still debated, with poor knowledge of the downstream signaling events controlled by Tau expression. We previously showed that several GBM cell lines do express the Tau protein, and among them, the U87-MG cell line at the highest level. The down-regulation of Tau expression by an RNA interference (shRNA) approach affected 2D-cell motility by inducing an inefficient cell-tail retraction. These data highlight a role for Tau as a crucial adaptor protein in the remodeling of MTs and actin networks and tuning cell motility, which is central in cancer cell invasiveness [20,21]. Speculating that a defective migratory ability may reveal a less aggressive behavior, here we used this cell model to study Tau’s involvement in GBM progression and to decipher the downstream signaling pathways.

We evaluated the contribution of Tau to GBM tumorigenicity in vivo, and we used Multi Cellular Spheroids (MCS) as a 3D human GBM model. The depletion of Tau in GBM cells delayed tumor progression in nude mice, inhibited in vitro spheroid growth and hindered cell evasion from spheroids. These phenotypes were accompanied by decreased MCS compactness, as revealed by an increase in MCS paracellular permeability, associated with altered expression and localization of N-cadherin. Moreover, a significant reduction of the phosphorylation state of Akt kinase revealed a faulty PI3K/Akt signaling pathway in GBM Tau-depleted cells. Accordingly, treatment of GBM control cells organized in MCS with the PI3K inhibitor LY294002 significantly inhibited MCS growth, while the Akt specific inhibitor, Perifosine, reduced MCS cell evasion. These findings were supported by in silico analysis of the human GBM tumors database (TCGA), confirming a positive correlation between Akt phosphorylation and MAPT gene expression. Taken together, our results highlight a new role for Tau in GBM cell 3D organization, growth and migration via the PI3K/Akt signaling axis.

## 2. Materials and Methods

### 2.1. Cell Culture

The generation and characterization of the U87shctrl and U87shTau cell clones by the shRNA approach was previously described [20]. Cells were maintained by the regular passage in a complete medium composed of Eagle’s minimum essential medium (EMEM, Lonza), supplemented with 10% fetal bovine serum (FBS, Lonza, Basel, Switzerland) and 2 mM of L-glutamine (Invitrogen, Thermo Fisher Scientific, Waltham, MA, USA) at 37 °C in a 5% CO_2_ atmosphere.

### 2.2. Multicellular Spheroid (MCS) Growth Assay

Adherent cells were detached by Trypsin-EDTA 0.05% (Thermo Fisher Scientific Waltham, MA, USA), counted and resuspended in a solution of 20% Methylcellulose in EMEM complete medium (2.5 × 10^4^ cells per mL). Then the cells were plated in 96-well suspension culture U bottom plates (ref.650185 Greiner Bio-one, Courtaboeuf CEDEX, France) (2500 cells per well). MCS were left to grow for up to 14 days in complete EMEM medium. The medium was renewed every three days. Spheroid growth was observed with a wide-field *CKX41* inverted microscope supplied with a 4× dry objective and equipped with a SC30 Color Camera (Olympus, Tokyo, Japan). Alternatively, an Eclipse TE 2000 (Nikon Instruments Inc., Melville, NY, USA), provided with a motorized stage equipped with a CCD camera CoolSNAP HQ (Photometrics, Tucson, AZ, USA), driven by NIS elements AR 2.30 software (Nikon), was used. One image per spheroid was taken every two days for up to 14 days. The area occupied by the spheroid was measured from images by Image J software and expressed as the percentage of the spheroid area at day 1 (mean ± SD, *n* = 8 spheroids). The experiments were repeated at least three times.

For treatments with inhibitors, twenty-four hours after spheroid formation (D0 of treatment), MCS were untreated (NT) or treated with Perifosine (Sigma-Aldrich, St. Louis, MO, USA, 5–10 µM in complete EMEM) or LY294002 (Sigma-Aldrich, St. Louis, MO, USA, 5–10 µM) for 2 days. Images of untreated and treated spheroids were taken at day 0 (D0) and day 2 (D2), and the area occupied by untreated and treated spheroids was measured by Image J software. Spheroid area values at D2 were expressed as the percentage of untreated spheroid area occupied at D0 (mean ± SD, *n* = 8 spheroids). The experiments were repeated at least three times.

### 2.3. MCS Cell Evasion Assay

Newly formed MCS (2500 cells) were settled on fibronectin extracellular matrix (10 µg/mL in phosphate buffer saline, PBS 1×). Images of evading cells from spheroids were taken by time-lapse video microscopy with a wide-field inverted microscope (obj dry 4× and 2× Eclipse TE 2000, Nikon Instruments Inc., Melville, NY, USA), provided with a motorized stage and connected with a CCD camera CoolSNAP HQ, driven by NIS elements AR 2.30 software. The microscope system was provided with a 37 °C incubation chamber and a CO_2_ and humidity control system. One frame was taken every hour (h) for 24 h. Quantification of cell evasion was performed from images taken at 0, 3, 6, 9 and 24 h. The extent of cell evasion from MCS was expressed as the following: (area of cell evasion at each time point-spheroid area at time 0 h)/ spheroid area at time 0 h; mean ± SEM, *n* = 4 spheroids). The cumulated results were from four experiments. For treatments, the inhibitors Perifosine and LY294002 (10 µM in complete EMEM medium) were added to the well, and then spheroids were settled on fibronectin, as described above. Monitoring and analysis of the evasion extent were performed as described above. The cumulated results are from three experiments.

### 2.4. MCS Paracellular Permeability Assay

Newly formed MCS (2500 cells, 24 h incubation) in methylcellulose were incubated with Dextran4000-FITC (0.25 µg/mL final concentration) for 2 h. MCS then were transferred onto fibronectin-coated 8-well chambered borosilicate coverglass Lab-Tek™ (Thermo Fisher Scientific, Waltham, MA, USA) and let to adhere for 30 min. MCS were observed with a Leica SP5 confocal laser scanning microscope (CLSM) equipped with a 20× (NA = 0.70) Oil PLAN APOCHROMAT objective. Optical sections of z = 1.98 µm from the bottom (adherent to slide) to the top of the spheroid were recorded. A total of 40–50 sections per spheroids were acquired. The quantification of Dextran-FITC infiltration through spheroid was measured by using Image J 2.1.0/1.53f51 software. For each optical section of the image stack of the spheroid: (i) the image was thresholded to have a binary image and was saved as image 1. (ii) the image was further processed by “Process-binary-fill holes” and saved as a mask, corresponding to the total area of spheroid section; (iii) image 1 was then subtracted from the mask using “Process image calculator-difference”, to obtain the final image showing infiltrating dextran–FITC area. “Analyze particle” was performed on the final image and the mask. The results were expressed as a ratio of the area particles (final image, area of FITC dextran infiltration)/area section (mask, total spheroid section area). The ratio values were expressed as a percent of the spheroid optical section having the highest ratio normalized to 1.

### 2.5. Immunostaining of Frozen MCS Sections and Confocal Microscopy Analysis

Newly formed MCS (10^5^ cells, 48 h incubation) were collected by centrifugation (about 40 MCS per condition), washed in PBS, mounted in a small cryomold in Optimal Cutting Temperature compound (Tissue-Tek^®^ OCT^™^ Compound, Sakura, Torrance, CA, USA) and frozen in liquid nitrogen vapor and stored at −80 °C. The frozen sections were obtained from OCT-included MCS (10 µm sections, temperature −23 °C) and settled on Superfrost + microscope slides (J1800AMNZ Thermo Fisher Scientific, Waltham, MA, USA). The sections were dried, fixed with methanol at −20 °C for 5 min, dried again and blocked for 15 min in PBS, BSA 5%, Saponin 0.05%. Primary antibodies, diluted in PBS, BSA 5%, Saponin 0.05% were added for 1 h incubation at RT. After washing in PBS, fluorochrome-coupled secondary antibody was added for overnight incubation at 4 °C in darkness. The antibodies used for immunostaining were; primary, anti-N-cadherin (rabbit polyclonal ab18203, targeting residues 800–900, Abcam, Cambridge, UK), anti-β-catenin (mouse monoclonal GC19220-50 BD, Transduction laboratories, Franklin Lakes, NJ, USA), anti-β-actin (mouse monoclonal AC-15 Novus, Saint Charles, MO, USA); secondary, goat anti-rabbit (Fab’)-AlexaFluor488 (A-11070 Thermo Fisher Scientific, Waltham, MA, USA), goat anti-mouse IgG-Alexafluor546 (A-11030 Thermo Fisher Scientific, Waltham, MA, USA). DRAQ5^TM^ (Thermo Fisher Scientific, Waltham, MA, USA) was used to counterstain nuclei. After washing, MCS were mounted in Prolong antifade medium (P36394, Thermo Fisher Scientific, Waltham, MA, USA). Images were acquired using a Leica SP5 confocal laser scanning microscope (CLSM) equipped with a 20× (NA: 0.70) and 63× (NA:1.4) Oil PLAN APOCHROMAT objectives (Leica microsystem, Weitzlar, Germany). A numerical zoom was applied to have 2.5× and 5× images. Changes in the N-cadherin staining distribution were quantified by analyzing the fluorescence intensity profiles (in arbitrary units, A.U.) along lines crossing cell–cell junctions (at least 30 lines by conditions). The results are expressed as fluorescence intensity as a function of distance (µm), using Image J 2.1.0/1.53f51 software, as described by Silvani G. et al. [22]. The width at the basis of the fluorescence-intensity peaks obtained from three independent experiments was then measured (*N* = 32 junctions) and plotted for each experimental condition. Analysis of co-localized fluorescence intensity on MCS double-immunolabeled sections was performed by measuring the R co-localization using the Pearson coefficient, with Image J 2.1.0/1.53f51 software, and “coloc-threshold” and “co-loc2” plug-ins.

### 2.6. MCS Viability Assay

Spheroids (2500 cells) were formed as described above. Twenty-four hours after spheroid formation (D0), MCS were untreated (NT) or treated with Akt inhibitor Perifosine (P. 5–10 µM in EMEM medium) or PI3K inhibitor LY294002 (LY. 5–10 µM) and kept in culture for up to 7 days. The medium and treatment were refreshed at days 2 (D2) and 5 (D5). At day 2 (D2) the EMEM complete medium containing MTT (3- [4,5- dimethylthiazol-2-yl]-2,5-diphenyl tetrazolium bromide) was added to each well at (final concentration 0.5 mg/mL) and incubated at 37 °C overnight, as previously described [23]. MCS were lysed, and the formazan solubilized using pure DMSO. The optical density (OD) was measured at 600 nm in a plate reader (Multiskan RC, Labsystems). The data were expressed as a percentage of survival of untreated U87shctrl cells set as 100% (mean ± SD, *n* = 8 spheroid). The experiments were repeated at least three times.

### 2.7. Western Blot Analysis

Cells or MCS were lysed in Pathscan lysis buffer (#7018, Cell Signaling Technology, Danvers, MA, USA): 20 mM Tris-HCl pH 7.5, 150 mM NaCl, 1 mM disodium EDTA, 1 mM EGTA, 1% Triton, 20 mM sodium pyrophosphate, 25 mM sodium fluoride, 1 mM b-glycerophosphate, 1 mM Na3VO4, 1 µg/mL leupeptin with Halt proteases inhibitor cocktail (Thermo Fisher Scientific, Waltham, MA, USA). Incubation was performed on ice for 30 min (min) with agitation. Lysates were then centrifuged at 13,000× *g* for 3 min at 4 °C. Equivalent amounts of proteins from the supernatant fraction were subjected to western blot analysis. Protein samples were loaded (30 μg/lane) and separated on 10% sodium dodecyl sulfate-polyacrylamide gels. The separated proteins were electrophoretically transferred onto Nitrocellulose Blotting Membrane (Amersham Protan, GE Healthcare, Chicago, IL, USA) using the Bio-Rad transfer system. The membranes were incubated with blocking solution for 1 h and then incubated overnight with the proper primary antibodies. The membranes were then washed 3 times with a PBST (PBS plus 0.05% Tween20) solution and incubated with horseradish-peroxidase-conjugated secondary antibodies for 1 h. The membranes were again washed 3 times with PBST and revealed using chemiluminescence HRP substrate (Merck) and the G-Box (Syngene). The following primary antibodies were used: anti-Tau (mouse Mab, ref. T-1029, USBiological, Salem, MA, USA), anti-Akt (rabbit polyclonal #9272, Cell Signaling Technology, Danvers, MA, USA), anti-phospho-Akt (Ser473) (rabbit polyclonal, # 9271, Cell Signaling Technology, Danvers, MA, USA), anti-phospho-Akt (Thr308) (rabbit Mab #13038, Cell Signaling Technology, Danvers, MA, USA), anti-N-cadherin (rabbit polyclonal ab18203, targeting residues 800–900, Abcam, Cambridge, UK), anti-β-catenin (mouse Mab GC19220-50 BD Transduction laboratories, Franklin Lakes, NJ, USA ), anti-β-actin (mouse Mab AC-15 Novus, Saint Charles, MO, USA ), anti-GAPDH (mouse Mab ref. G8795, Sigma-Aldrich, Merck, St. Louis, MO, USA). The HRP-coupled secondary antibodies were purchased from Cell Signaling Technology. Densitometric analysis of band intensities was performed using the NIH ImageJ software.

### 2.8. Intracellular Pathscan Signalling Array

The PathScan Intracellular signaling array kit from Cell Signaling Technology (#7323 Cell signaling Technology, Danvers, MA, USA) was used to investigate the modification of the signaling pathway activation, as previously described [23]. Briefly, cells were lysed in pathscan lysis buffer (20 mM Tris-HCl pH 7.5, 150 mM NaCl, 1 mM disodium EDTA, 1 mM EGTA, 1% Triton, 20 mM sodium pyrophosphate, 25 mM sodium fluoride, 1 mM b-glycerophosphate, 1 mM Na3VO4, 1 µg/mL leupeptin added of Halt proteases inhibitor cocktail (Thermo Fisher Scientific, Waltham, MA, USA). The lysates were centrifuged at 13,000 *g* at 4 °C. The supernatant was collected, and the proteins were quantified by the Bradford protein assay (Biorad, Hercules, CA, USA). The samples were diluted in lysis buffer to obtain a final concentration of 1.2 mg/mL in 50 μL. The slides were saturated for 15 min with the saturation buffer provided in the kit and 50 μL of the lysates were added to the different wells. After overnight incubation at 4 °C, the wells were washed for 5 min three times. The slides were incubated with the detection antibody provided in the kit for 1 h at room temperature. After washing, the slides were treated with HRP-coupled streptavidin (provided in the kit) for 30 min at room temperature. After another series of washes, the chemiluminescence was revealed using the Lumiglo/peroxide detection kit (provided by the manufacturer) and observed with the Syngene G-Box. The intensity of the chemiluminescence was quantified with ImageJ 2.1.0/1.53f51 software. The integrated density values, after subtraction of the intensity of the negative control, were expressed as a percentage, using the kit positive control mean as 100% (mean ± SD). The values of the phosphorylation levels of Akt Residues Ser473 and Thr308 were expressed as a ratio on U87shctrl set as 1.

### 2.9. In Vivo Experiments

All experimental procedures and animal care were carried out in conformity with the guidelines of the French Government and approved by the Regional Committee for Ethics on Animal Experiments (Marseille number 14, authorization number 2016073009409029). Six to 8 weeks old female athymic nude mice were obtained from Charles River Laboratories France (L’Arbresle, FR). After animal anesthesia, 5 × 10^5^ U87shctrl or U87shTau cells were injected into the corpus callosum (1 mm anterior to bregma, −1mm lateral and −2 mm in deep of the cortex surface) as previously described [24,25]. The animals were observed until they fully recovered. The body weight and clinical status of the mice were recorded every 2 days. The mice were euthanized when they exhibited more than a 20% reduction from initial body weight or significant neurological deficit. Brains were extracted, fixed in formalin and paraffin-embedded according to standard procedures. The paraffin-embedded brains were serially sectioned using a microtome, and 5 µm coronal sections were stained with Haematoxylin and Eosin (HE), as previously described [24,25]. Images of the HE-stained brain sections were acquired with a slide scanner (Hamamatsu photonics, Hamamatsu City, Japan). The analysis of tumor size at the largest brain sections was performed as previously described [26], using Image J software. The results were expressed as the ratio of tumor area over total brain area (%).

### 2.10. TCGA Dataset Analysis

We used the available public cohort of human glioblastomas from The Cancer Genome Atlas (TCGA, https://tcga-data.nci.nih.gov/tcga/, accessed on 31 May 2021) and proteomic data from The Cancer Proteome Atlas (TCPA, https://tcpaportal.org/tcpa/, accessed on 31 May 2021) [27]. Reverse-phase protein array (RPPA_AnnotateWithGene.Level_3), clinical annotation (Merge_Clinical. Level_1) and gene expression of the glioblastoma TCGA dataset (Merge_transcriptome__ht_hg_u133a__broad_mit_edu__Level_3__gene_rma__data.Level_3) were retrieved using the RTCGA package (https://rdrr.io/bioc/RTCGA/, accessed on 31 May 2021) and R Studio. Tumors (*N* = 158) were stratified into two groups based on the Tau (*MAPT)* RNA expression values below (low) and above (high) the median values (Affymetrix HG-U133A) [27,28,29].

### 2.11. Statistical Analysis

The data are presented as mean ± SEM. Statistical significance was tested using unpaired Student’s T-test or one-way ANOVA when more than two groups were compared. For experiments using multiple variables, statistical significance was assessed via two-way ANOVA with Bonferroni post-test correction. All experiments were repeated at least three times except for the PathScan array and for in vivo experiments. Asterisks indicate significant difference between conditions: * *p*  <  0.05; ** *p*  <  0.01; *** *p*  <  0.001. Statistical analyses were performed with PRISM GraphPad 5.0 statistical software and Excel software.

## 3. Results

### 3.1. Tau Down-Regulation Reduces In Vivo Glioblastoma Tumorigenicity

**Figure 1:**
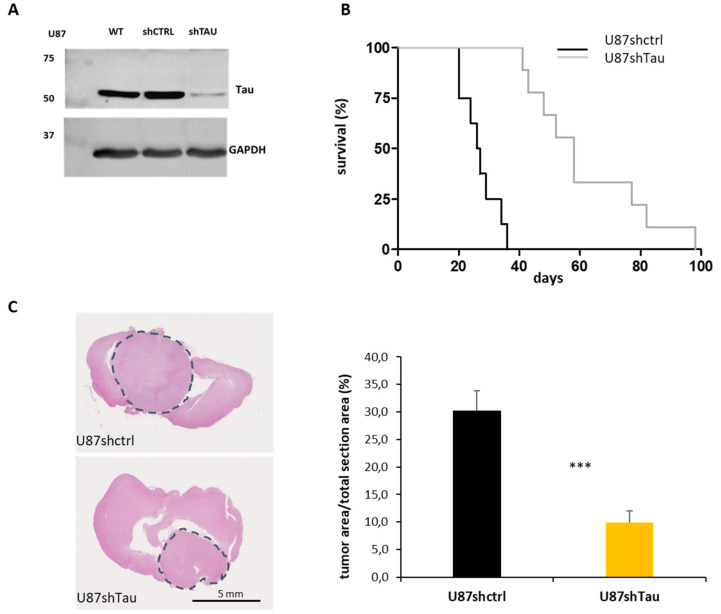
Tau down-regulation reduces in vivo tumorigenicity. (**A**) Western blot analysis for Tau expression of U87 wild-type (WT), shctrl and shTau cell total protein lysates (30 µg). GAPDH was used as the loading control. (**B**) Kaplan–Meier survival plot of *nude* mice intracranially grafted with U87shctrl and U87shTau cells. (*N* = 9 mice, for each group). (**C**) Histological analysis of brains from mice intracerebrally grafted with U87shctrl and U87shTau cells (H.E. staining). Left: representative images of coronal sections, the grey circles indicate tumor tissue. Right: quantification of the area occupied by the tumor at the maximal brain tumor dimensions in the sections. Results are expressed as the percentage of apparent tumor area on brain area section (*N* = 8 mice for each group, *** *p* < 0.001).

We first investigated the role of Tau in high-grade gliomas. For this purpose, *nude* mice were orthotopically engrafted with U87 stable cell clones expressing endogenous levels (U87shctrl) or reduced levels of Tau (U87shTau) (see Figure 1A). Mice injected with U87shTau cells showed a significantly longer survival (median survival 58 days) compared to mice injected with U87shctrl cells (median survival 26.5 days), as shown by Kaplan–Meier analysis (Figure 1B, *p* < 0.001). The histological analysis of post-mortem mice brain sections showed that the average tumor area occupied over the total brain area at the time of death was significantly lower in mice injected with U87shTau cells compared to mice injected with U87shctrl cells (Figure 1C: representative images and quantification from images: 9.2 ± 2.2% for U87shTau tumor sections vs. 30.2 ± 3.7% of the total area for U87shctrl tumor sections, *p* < 0.001). These results indicate that Tau contributes to tumor progression, possibly through the regulation of cell growth and/or invasion.

### 3.2. Tau Down-Regulation Hinders Multicellular Spheroid Growth and Cell Evasion

**Figure 2:**
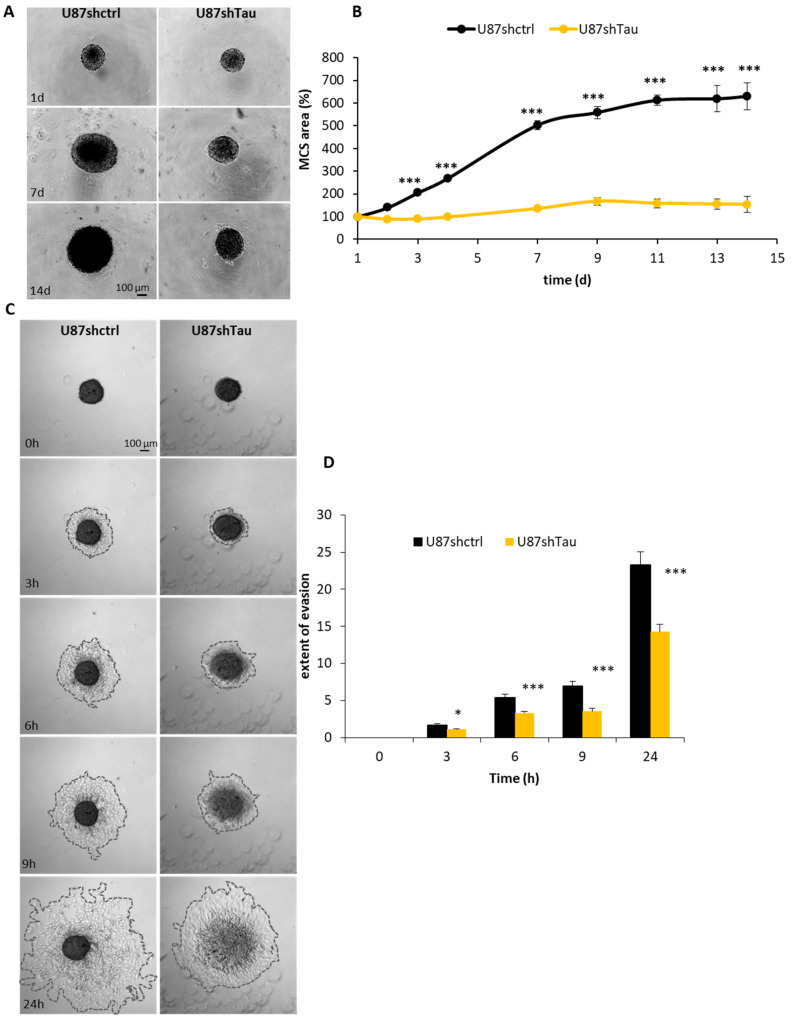
Tau knockdown hinders multicellular spheroid growth and cell evasion. (**A**) Time course of MCS growth. Spheroids were grown in 20% methylcellulose for up to 15 days. Representative images of spheroids at 1, 7 and 14 days (d) are shown. (**B**) Quantification of the apparent area occupied by spheroid. Values are expressed as % of area occupied by spheroid at day (d) 1 (*N* = 8, *** *p* > 0.001, U87shctrl vs. U87shTau at each time point). (**C**). MCS were set on a fibronectin matrix and evasion analyzed by time-lapse video microscopy. Representative images at 3-6-9-24 hours (h) are shown. (**D**) Quantification of the extent of MCS evasion. Results are expressed as: (area of cell evasion at each time point-spheroid area at time 0 h)/spheroid area at time 0 h, *N* = 16, * *p* < 0.05, *** *p* < 0.001, U87shctrl vs. U87shTau at each time point).

To better understand how Tau can favor tumor progression, we evaluated the effect of Tau down-regulation on MCS growth, a well-characterized 3D tumor cell model [29]. We measured, on microscopic images, the apparent area occupied by spheroids over time. U87shctrl spheroids grew over time up to 6-folds of their starting area. In contrast, U87shTau MCS grew significantly more slowly starting from day 2 (154 ± 10% U87shTau vs. 630 ± 61% U87shctrl, *p* < 0.001 at day 14, Figure 2A,B, for quantification). This phenomenon was not due to a defect in the initial spheroid formation since the time course of cell aggregation into spheroid was similar for both U87shTau and U87shctrl cells (Supplementary Figure S1). The difference in MCS growth rate was likely due to a slowing down of U87shTau cells proliferation rate, since their doubling time was 31 ± 0.8 h while U87shctrl cells doubling time was 22 ± 0.4 h, (*p* < 0.001, Figure S2A,B). However, Tau depletion did not significantly modify the cell area and/or volume. Indeed, the number of cell nuclei per 100 µm^2^ of area spheroid section was not significantly different in U87shctrl and U87shTau spheroids (Figure S2C).

We next evaluated whether Tau regulates cell migration in a 3D system. To this end, MCS were plated on a fibronectin-coated surface, and cell evasion was monitored by time-lapse video microscopy. U87shctrl and U87shTau cells migrated away from pre-formed spheroids over time. However, as early as 3 h, the extent of cell evasion from U87shTau spheroids was significantly reduced compared to that from U87shctrl spheroids (Figure 2C,D, for quantification and movies S1 and S2). The area covered by evading cells was 1.7 ± 0.2-fold the area at time 0 h for U87shctrl, while it was 1.1 ± 0.1-fold for U87shTau (*p* < 0.05). This difference was even greater at later time points (at 24 h: 23.3 ± 1.9 folds the area at time 0 h for U87shctrl vs. 14.3 ± 1.1 folds the area at time 0 h for U87shTau, *p* < 0.001), indicating that U87shTau cells migrated less from the spheroid core compared to U87shctrl cells (Figure 2B). Of note, despite their similar appearance at time 0 h, U87shctrl core spheroids did not change in size and morphology over time, while U87shTau core spheroids disassembled after 6–9 h (Figure 2B and movies S1 and S2). We thus wondered if the lower ability of U87shTau cells to evade was due to a defective cell adhesion to ECM. As shown in Supplementary Figure S3, the percentage of cells able to adhere to fibronectin, collagen I and vitronectin matrices was similar for both cell types, excluding any significant defect of U87shTau cells adhesive properties as compared to U87shctrl cells. In addition, we can also exclude an effect of differential proliferation in our assays, which were performed over a shorter time frame than cell doubling times (Supplementary Figure S2A,B).

Altogether, these results show that Tau promotes long-term spheroid growth and cell evasion by impacting not only cell migration but also spheroid cohesion.

### 3.3. Tau Down-Regulation Decreases Multicellular Spheroid Paracellular Permeability and Induces N-Cadherin Mislocalization

**Figure 3:**
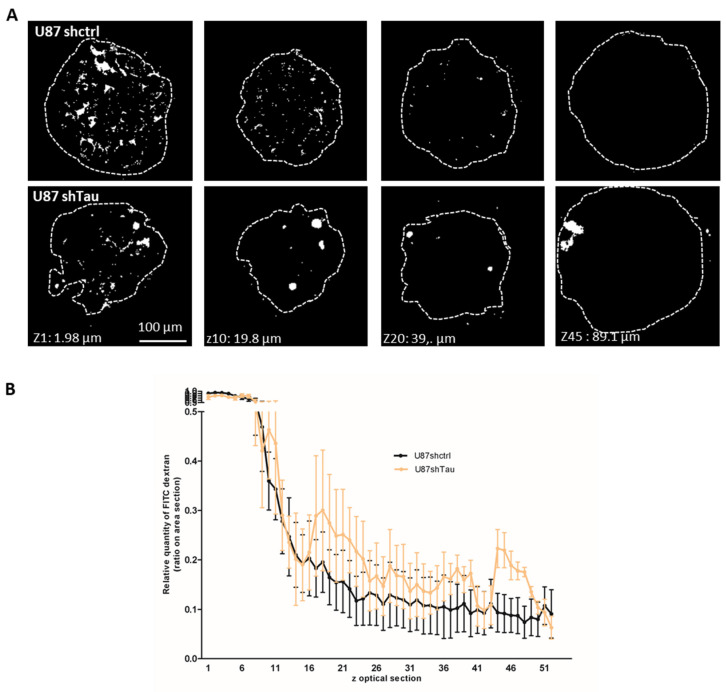
Tau down-regulation affects spheroid paracellular permeability. (**A**) Spheroids were incubated with FITC-Dextran for 1 h and settled on fibronectin matrix for 30 min before confocal microscopy analysis. Representative images of z optical sections (*z* = 1.98 µm) after image thresholding are shown (obj 20×). (**B**) Quantification of FITC-Dextran infiltration in spheroids. The graph shows the area of FITC staining through spheroid optical sections. The relative quantity of FITC-Dextran accumulation is expressed as the ratio of the area of FITC-labeled objects on the total area of spheroid optical sections, expressed as a percent of sections having the highest FITC accumulation normalized to 1 (*N* = 6 for each group MCS, *p* < 0.001).

Cell cohesion is important to ensure spheroid compaction and requires fine-tuning of cell–cell interactions inside the spheroid [29]. We compared the compactness of U87shctrl and U87shTau MCS through the measurement of paracellular permeability. This assay consists of testing the ability of spheroid cells to exclude FITC-Dextran, which does not penetrate through cell membrane but diffuses and accumulates in intercellular spaces according to cell-ell contact tightness [30]. As observed by confocal microscopy, FITC-Dextran accumulated in the intercellular space in the first 10 optical sections of the U87shctrl spheroid, starting from the adherent bottom. This accumulation of FITC-Dextran dropped out with spheroid depth in U87shctrl MCS, starting from optical section z15, suggesting a decrease in paracellular permeability (Figure 3A, representative images). Dye accumulation was not different in the first 15 optical sections of U87shTau MCS. However, FITC-Dextran still accumulated in intercellular spaces of z20–50 optical sections of U87shTau MCS, forming “FITC-Dextran pockets” that were not observed in U87shctrl spheroids (Figure 3A, representative images). A quantification from the serial sections showed that the intercellular accumulation of the dye was significantly higher for U87shTau compared to U87shctrl MCS in Z20–50 sections (Figure 3B, *p <* 0.001). These results suggest that Tau may be implicated in maintaining MCS compactness, as revealed by the increase in spheroid paracellular permeability in Tau-depleted MCS.

**Figure 4:**
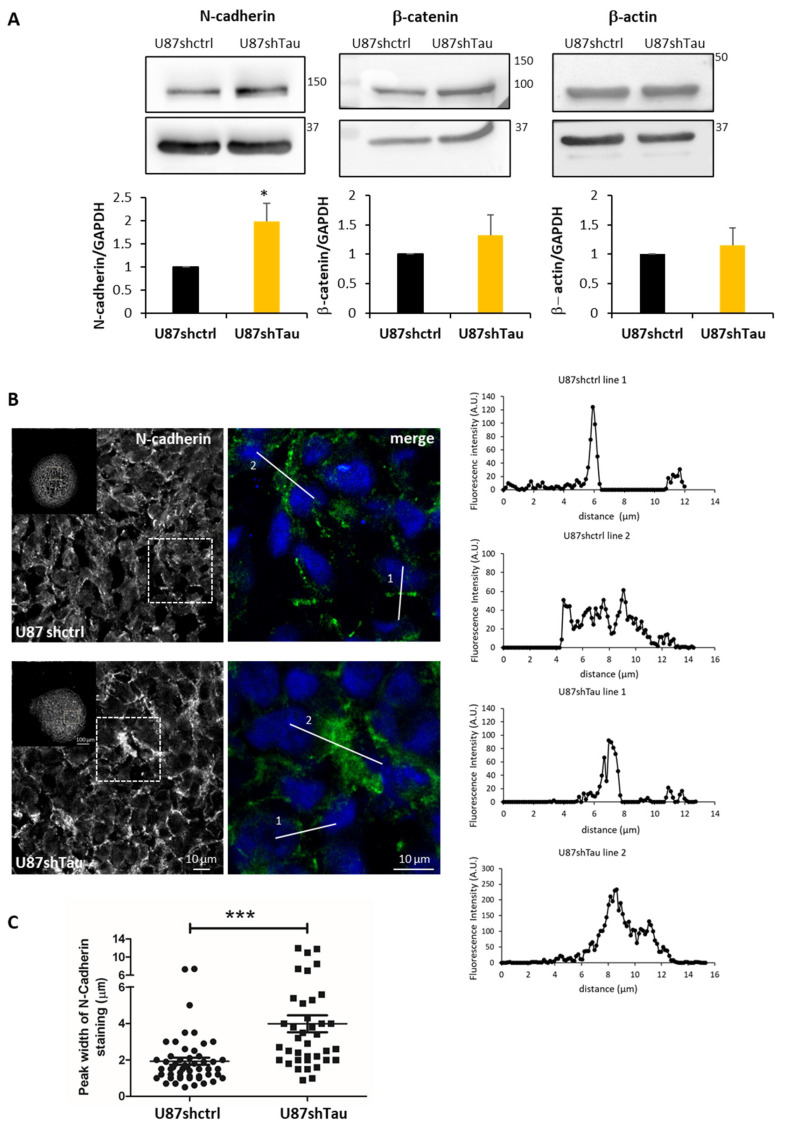
Tau down-regulation modifies N-cadherin localization at the cell–cell contacts in MCS. (**A**) Upper panel: western blot analysis of N-cadherin, β-catenin and β-actin expression in MCS total protein lysates (25 µg, GAPDH was used as the loading control). Lower panel: densitometric analysis of band intensities; *N* = 3, * *p* < 0.05). (**B**) Confocal microscopy images showing N-cadherin localization in MCS. Left: one optical section from a 10 frames stack, obj 20×, 5× magnification of the 1x image in the small insert. Right: merged image with nuclei. Lines are representative scans across the cell–cell borders along which fluorescence intensity profiles were obtained. Graphs on the right show the fluorescence intensity profiles (A.U.) as a function of distance. (**C**) Quantification of the peak width of N-cadherin staining (µm) from fluorescence intensity profiles (*N* = 32 *** *p* < 0.001).

Cell junction molecules, such as cadherins, are responsible for cell–cell adhesion [29]. We analyzed the expression of N-cadherin, its partner β-Catenin, and β-actin. Protein extracts from U87shTau spheroids expressed significantly higher levels of N-cadherin compared to U87shctrl, as analyzed by Western Blot, while the expression of β-catenin and β-actin were not significantly increased (Figure 4A). We next performed the immunostaining of N-cadherin on MCS cryosections and analyzed them by confocal microscopy. Both U87shctrl and U87shTau spheroid sections expressed N-cadherin at the cell–cell junctions (Figure 4B). However, N-cadherin staining in U87shctrl spheroids appeared discontinuous with a punctate distribution, compatible with a localization at the cell membrane, while the staining appeared stronger and more diffuse in U87shTau MCS cells (Figure 4B, see lower panel for higher magnification). To better characterize the thickening of N-cadherin staining, we analyzed the fluorescence intensity profiles along lines crossing cell–cell junctions (two representative profiles for each condition are shown on the right panels of Figure 4B). In both U87hctrl and U87shTau sections’ thin peak fluorescence profiles, corresponding to localized N-cadherin staining, and larges peaks, corresponding to more diffuse staining were observed. The quantification revealed that the mean (±SEM) peak width was significantly increased in U87shTau MCS sections (Figure 4C) compared to the control, suggesting that Tau depletion results in a more diffuse distribution of N-Cadherin at cell–cell junctions.

**Figure 5:**
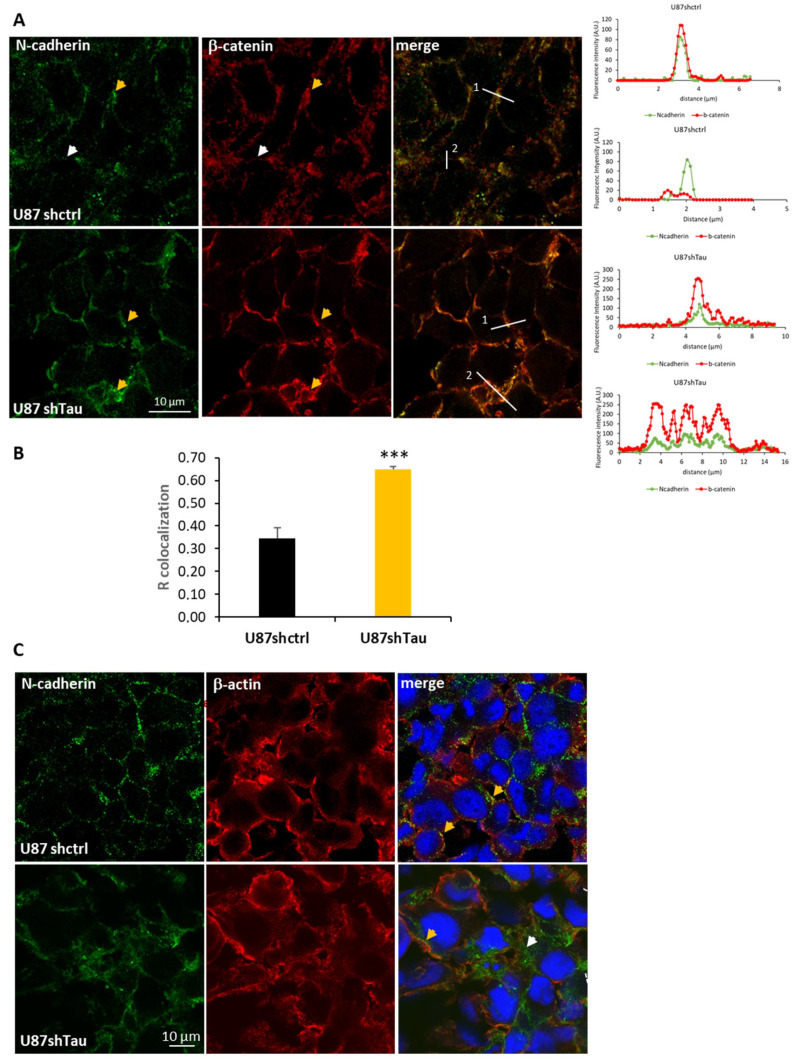
Tau down-regulation modifies N-cadherin and β-catenin but not β-actin localization. (**A**) Left panel: Confocal microscopy images showing localization of N-cadherin (green) and β-catenin (red, obj 63×, 1 section from a 10 frames stack, numerical zoom 2.5×); yellow arrowheads indicate co-localization, and white arrowheads indicate lack of co-localization. Right panels: representative fluorescence intensity profiles (A.U.) obtained along lines indicated on images. (**B**) Quantification of co-localization (R co-localization, Pearson coefficient) of fluorescence signal in MCS sections (*N* = 12 *** *p* < 0.001). (**C**) Confocal microscopy images showing localization of N-cadherin (green), β-actin (red) and merged signal with nuclei (blue, obj 63×, 1 section from a 10 frames stack, numerical zoom 2.5×); yellow arrowheads point to junctional staining of N-cadherin, next to β-actin, white arrowhead indicates diffuse N-cadherin staining.

Intracellular domains of N-cadherin dimers recruit p120, β-catenin and α-catenin, which link the complex to the actin filaments of the cytoskeleton [31]. To better characterize the localization of the N-cadherin-β-catenin junctional complex, we performed double immunostaining. In U87shctrl spheroids, a large proportion of N-cadherin co-localized with β-catenin in discrete clusters as expected [32,33], while a fraction of N-cadherin did not (Figure 5A left, yellow and with arrowheads, and right, line scans profiles). In contrast, N-cadherin and β-catenin appeared almost always co-localized at the cell–cell borders of U87shTau spheroids in discrete clusters and in more diffuse regions (Figure 5A left, yellow arrowheads, and right, line scans profiles). Accordingly, the measure of co-localization Pearson coefficient (R co-localization) in MCS sections was significantly higher in U87shTau spheroids compared to U87shctrl (Figure 5B). Double staining of N-cadherin and cortical β-actin showed fluorescent signal proximity for N-cadherin and β-actin at the cell membrane of U87-shctrl cells suggesting a stabilization of the membrane N-cadherin to the actin cytoskeleton (Figure 5C, yellow arrowheads). Interestingly, in U87shTau MCS, this signal proximity for N-cadherin and β-actin was only observed in localized cortical regions, while the more diffuse staining did not (Figure 5C, yellow vs. white arrowheads). Therefore, the depletion of Tau induced a more diffuse localization of N-cadherin and β-catenin at cell–cell borders while not affecting β-actin localization. Interestingly, the analysis of N-cadherin expression and localization at the cell membrane was not affected by Tau down-regulation in 2D-cultured adherent cells (Supplementary Figure S4). These results suggest that Tau may be involved in the stabilization of N-cadherin to the actin cytoskeleton at the cell–cell contacts in MCS, linking Tau and N-Cadherin in 3D cell organization.

### 3.4. Tau Regulates Multicellular Spheroid Organization, Growth, and Migration via the PI3K-Akt Signalling Pathway

**Figure 6:**
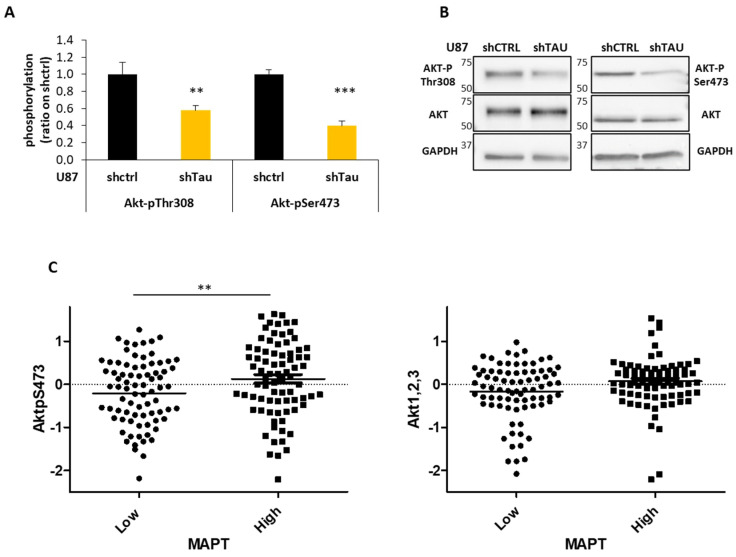
Tau expression correlates with activation of the PI3K-Akt signaling pathway in glioblastoma. (**A**) Pathscan assay. Quantification of the phosphorylation levels of Akt in total cell lysates; (*N* = 4, ** *p* < 0.01, *** *p* < 0.001 U87shTau versus U87shctrl). (**B**) Western blot analysis for Akt-pThr308, Akt-pSer473 and total Akt expression in total protein cell lysates (25 µg). GAPDH was used as the loading control. (**C**) Analysis of the amount of Akt-pSer473 and Akt 1,2,3 total protein from TCGA-GBM Reverse-phase Protein Array (RPPA) dataset. Tumors (*N* = 158) were stratified into two groups based on Tau (*MAPT)* RNA expression values below (low) and above (high) the median values (Affymetrix HG-U133A, ** *p* < 0.01).

The recruitment of cadherin–catenin complexes at the cell junctions initiates a variety of molecular events leading to the activation of intracellular signaling pathways [34]. To identify the signal transduction pathway implicated in Tau-regulated 3D cell organization, growth and evasion, we performed a PathScan^®^ Intracellular Signaling array, as previously described [23,35]. Among the different signaling molecules analyzed, Akt presented significantly lower levels of phosphorylation at both Ser473 (*p* < 0.001) and Thr308 (*p* < 0.01) residues in U87shTau compared to U87shctrl cell lysates (Figure 6A). This result was confirmed by immunoblot, suggesting that Akt activity may be lower in cells expressing low levels of Tau (Figure 6B). Indeed, these two residues are described to be phosphorylated in the active form of Akt kinase [36]. Interestingly, among the several key signaling pathways altered in glioblastoma, the PI3K/Akt pathway predominantly contributes to the pathology of the disease [37]. In agreement with our in vitro results, in silico analysis of Reverse-phase Protein Arrays (RPPA) data from a cohort of patients (TCGA-GBM dataset) showed a significant positive correlation between Tau expression and Akt activity since lower levels of AktSer473 phosphorylation were found in human glioblastoma expressing low levels of Tau RNA (*MAPT p* < 0.01). In contrast, the total level of Akt protein was similar between the two groups of patients (Figure 6C). These results show a close relationship between Tau expression and Akt activity in glioblastoma.

**Figure 7:**
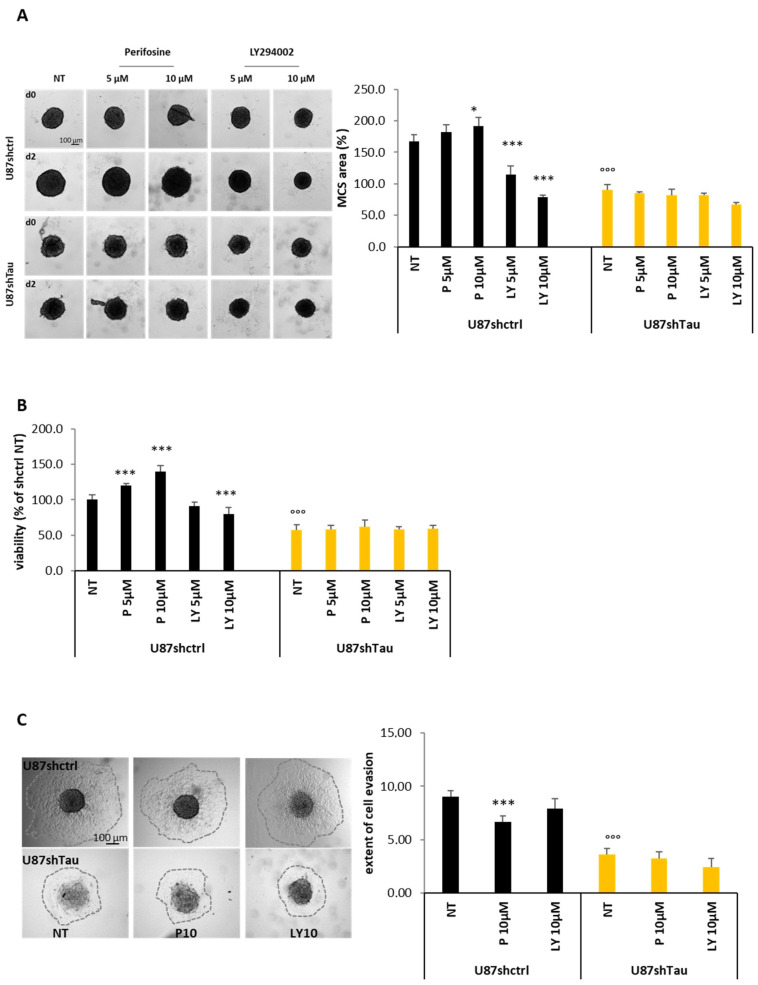
The PI3k-/Akt signaling pathway is differentially implicated in Tau-dependent MCS growth and evasion. (**A**) Effect of PI3-K and Akt inhibition on spheroids growth in 20% methylcellulose. On day 1 of spheroid formation (d0) spheroids were treated or not (NT) with Perifosine (P 5-10 µM) or LY294002 (LY 5–10 µM) for 2 days. Left: representative images are shown. Lower part: quantification of the apparent area occupied by MCS. Values are expressed as % of area occupied by MCS before treatment (D0; * *p* < 0.05, *** *p* < 0.001 U87shctrl NT vs. U87shctrl P10 µM, LY 5 µM and LY 10 µM, respectively; °°° *p* < 0.001 U87shctrl NT vs. U87shTau NT; *N* = 8). (**B**) Effect of Akt inhibition on spheroid cell viability (MTT assay). Values were expressed as % of NT U87shctrl condition (*** *p* < 0.001 U87shctrl NT vs. U87shctrl P5 P10 and LY 10 µM, respectively; °°° *p* < 0.001 U87shctrl NT vs. U87shTau NT, *N* = 8). (**C**) Effect of PI3K -Akt inhibition on cell evasion from spheroids. Left: representative images of evasion at time 9 h. Right: quantification of evasion. Results are expressed as the ratio of the area occupied by the migrating cells from the spheroid on the area of the spheroid at time 0 h (*N* = 12; *** *p* < 0.001 U87shctrl vs. U87shctrl P10 µM; °°° *p* < 0.001 U87shctrl NT vs. U87shTau NT).

To confirm the relationship between Tau and the PI3K/Akt pathway, we next evaluated if the inhibition of the PI3K/Akt activity may affect spheroid growth and evasion in U87shctrl and U87shTau cells. To this end, we treated spheroids with increasing concentrations of the PI3K-Akt inhibitor LY294002 and the specific Akt inhibitor Perifosine (5–10 µM), and we measured their apparent area occupied, as before. We confirmed that the area of the untreated U87shTau spheroid was significantly reduced compared to that of untreated U87shctrl spheroids (reduction of 50% of U87shctrl area after two days treatment (D2) relative to day 0 (D0, *p* < 0,001, Figure 7A). Treatment with LY294002 significantly reduced U87shctrl spheroid size in a dose-dependent manner, recapitulating the phenotype of Tau-deficient spheroids (reduction of 32 and 53% at 5 and 10 µM respectively, *p* < 0.001). Perifosine did not reduce U87shctrl spheroid size but rather increased their apparent area. None of the treatments significantly affected the size of U87shTau spheroids (Figure 7A). As an orthogonal approach, we tested MCS viability using MTT assay and obtained similar results. The U87shTau spheroid presented 40% less viability than the U87shctrl spheroid according to their reduced size (Figure 7B). The viability of U87shctrl spheroids was reduced in a dose-dependent manner by LY294002 (reduction of 20% at 10 µM, *p* < 0.001), while it was increased by Perifosine treatment (+20 and +40% at 5 and 10 µM, respectively; *p* < 0.001). Here again, none of the treatments affected the MTT signals obtained with U87shTau spheroids (Figure 7B). Therefore, the inhibition of PI3K, which is upstream of Akt, was effective in hindering spheroid growth and survival, while Akt inhibition did not.

The above results suggest that higher AktSer473 phosphorylation observed in patient samples expressing high levels of MAPT may be not correlated to tumor growth but rather reflect differences in cell behavior, such as cell–cell adhesion or cell migration. To test such a possibility, we thus performed evasion assays from MCS in the presence of Perifosine or LY294002 (10 µM). As already shown, the extent of evasion of U87shTau cells from the spheroid was significantly reduced compared to U87shctrl (Figure 7C, time 9 h is shown). Specific inhibition of Akt by Perifosine treatment significantly inhibited U87shctrl but not U87shTau cells evasion, while treatment with the PI3K inhibitor LY294002 did not affect cell evasion. All together, these results suggest a role for Tau as an upstream regulator of the PI3K/AKT pathway. Akt activity may be necessary for Tau-dependent cell migration, while upstream PI3K activity may be involved in the control of cell growth and survival.

## 4. Discussion

The protein Tau is a molecule at the crossroads between brain tumors and neurodegenerative diseases [17,38]. Tau accumulation and aberrant phosphorylation is a driver of Alzheimer’s disease, and Tau expression has also been recognized as a prognosis or predictive marker for chemotherapeutic response in several cancers [11,17,39]. In the present work, by down-regulating Tau expression in glioblastoma U87-MG cells, we show that Tau contributes to tumor progression in vivo and in vitro. Available published results suggest that the function of Tau in cancer is dependent on tumor/cell type [17]. Previous work has shown that *MAPT* gene expression is enriched in LGGs as compared to GBMs [19,40]. In particular, Tau expression positively correlates with overall patient survival in LGGs and GBMs subtypes carrying mutations in the Isocitrate Dehydrogenase 1/2 (*IDH1/2*) gene, which are considered to be of better prognosis [19,41]. It has been suggested that Tau expression is epigenetically induced and regulates the tumor microenvironment in IDH1/2 mutated tumors, resulting in vascular normalization and limited tumor progression [41]. The relationship between Tau expression and disease aggressiveness in wild-type *IDH1/2* GBMs remains less clear. Our experiments, based on a wild-type *IDH1/2* GBM cell line model, suggest a more direct role of Tau in tumor cell properties.

We found that Tau-depletion significantly inhibited in vitro spheroid growth and reduced 2D cell proliferation. This is in agreement with results showing that decreased Tau protein in breast cancer cells results in lower proliferation, decreased migration and reduced cell invasion [42]. However, such a function is not shared by all cell types and is likely dependent on microtubule composition and on the level of Tau expression [43,44]. For instance, in ovarian cancer, the down-regulation of Tau affected the viability of the high Tau-expressing TOV112V cell line while did not affect the low-Tau expressing OVCAR cell line (Yamauchi et al., 2017). It has also been proposed as a direct function of Tau in stabilizing DNA and/or mitotic spindle assembly, conferring a selective advantage for cancer cell growth [45,46].

We and others have previously shown that GBM-derived cells migration is an MT-dependent process characterized by the formation and extension of forward protrusions toward which the rest of the cell glides [5,47,48]. Tau depletion in U87 and U251 cells reduces 2D cell velocity and distance-to origin due to a delay in retraction of the back [20]. In the present work, we confirm its crucial role in directing cell migration and extended these data to a three-dimensional model.

Cell organization in 3D spheroids consists of an initial aggregation of isolated cells, followed by cadherin-dependent spheroid compaction and growth [49,50]. We showed that Tau depletion did not affect cell aggregation into spheroids but induced a diffuse localization of the N-cadherin-β-catenin complex at cell–cell borders. However, cortical actin localization was unchanged, suggesting an uncoupling from the actin cytoskeleton. Previously published work has shown that defective ependymal cell differentiation and organization in mouse brain was associated with disruption of adherent junctions and altered N-cadherin localization [51]. In agreement with this work, we can speculate that a defective recruitment and/or clustering of N-cadherin at the membrane might be responsible for the loss of spheroid compactness and increased paracellular permeability in U87shTau MCS [50,52]. Mislocalized N-cadherin may sequester β-catenin, resulting in an apparent increased co-localization of the proteins [51]. However, we cannot exclude that Tau depletion may also induce N-cadherin intracellular retention or increase of N-cadherin endocytosis. We can thus postulate that Tau stabilizes cadherin–catenin complexes to the actin cytoskeleton, favoring *trans* and *cis* adhesion molecules interactions. It has also been reported that an intact MT network is necessary to translocate N-cadherin to cell plasma membrane [53]. Therefore, the reduced expression of Tau may alternatively disorganize the MT-dependent cadherin–catenin trafficking, eventually compensated by an increased expression of N-cadherin.

Cadherin homophilic cell–cell adhesion activates pro-survival intracellular signaling pathways in cancer cells that involve the PI3K/Akt signaling cascade [54,55]. In gliomas, the abnormal behavior of cancer cells is mainly characterized by the constitutive activation of this signaling pathway, particularly in *PTEN* mutated/deleted GBMs. Interestingly, Tau expression has been inversely correlated to *PTEN* mutation/deletion in gliomas, prostate and breast cancer [16,41,56]. U87-MG cells carry a mutation in the *PTEN* gene intron leading to the loss of PTEN expression [3,57]. As a main result, we found that Tau depletion in our cell model was associated with reduced Akt activation, as demonstrated by reduced AktSer473 and AktThr308 phosphorylation. Interestingly, our analysis of publicly available TCGA human GBM datasets showed a positive association between phosphorylation of AktSer473 and *MAPT* (Tau) RNA expression, in agreement with our experimental results. This suggests that Tau may contribute to Akt activation in the context of PTEN loss of function. The prognostic value of AktSer473 for poor disease outcome has been underlined in several cancers, such as prostate cancer [58]. We could not find, by analysis of the TCGA GBM cohort, a significant negative correlation between the expression of phosphorylated AktSer473 and overall patient survival. This may be due to the limited number of TCGA samples for which proteomic data of Akt phosphorylation are available and underlines the need for refined studies on selected samples. However, a previous publication revealed a positive association between increased AktSer473 phosphorylation in patient tissue samples and progression from anaplastic astrocytoma to glioblastoma [59].

Of note, the specific inhibition of PI3K kinase, which is upstream of Akt, significantly reduced spheroid growth and survival in Tau-expressing cells, while specific Akt inhibition with Perifosine did not. This can be explained by the fact that other molecules than Akt and downstream of PI3K may be involved in tumor growth and cell survival [60,61]. These results suggest that the higher Akt Ser473 phosphorylation observed in patient samples expressing high levels of *MAPT* RNA may not be correlated to tumor growth but rather reflects differences in cell behavior, such as cell–cell adhesion or cell migration. This is supported by the finding that Akt inhibition with Perifosine significantly reduced MCS cell evasion of Tau-expressing cells while without further affecting the evasion of Tau-depleted cells. Such a result might appear counter-intuitive with the fact that PI3K inhibition did not affect cell migration irrespective of Tau expression. PI3K allows the recruitment of PDK1 and Akt to the plasma membrane and the consequent phosphorylation of Akt on Thr308 and Ser473 [62]. However, it has been shown that other kinases may activate Akt independently of PI3K [63]. Comforting our data, a relationship between Tau and the PI3K/Akt pathway has been previously proposed since the down-regulation of Tau in Docetaxel-resistant prostate cancer cells rescued drug resistance by inhibiting the PI3K/Akt/mTOR signaling pathway [13].

How may Tau regulate the PI3K/Akt signaling pathway? It has been shown that the recruitment of PI3K by the cadherin-catenin complex at the membrane initiates the signaling cascade [54,61]. Tau may, therefore, strengthen these molecular interactions via the stabilization of the MT network. Alternatively, we can postulate an MT-independent interaction of Tau with PI3K/Akt molecules, as previously reported for Fyn and Src kinases [64]. Indeed, a direct association of Tau to PI3K has been revealed in prostate cancer cells [65]. Thus, Tau may cooperate by interacting directly with PI3K in order to activate the Akt signaling pathway. Interestingly, a similar role in inducing the activation of the PI3K pathway in *PTEN*-deleted tumors has been shown for Stathmin, another key regulator of MT dynamics [66,67].

Targeting the PI3K/Akt pathway in glioblastoma therapy has been more and more investigated [68,69]. For instance, it has been shown that PI3K inhibitors, such as buparlisib, have synergistic activity in combination with Temozolomide in *PTEN*-null GBM mouse xenografts. Current clinical trials evaluate the efficacy of the therapeutic approach of PI3K/Akt inhibition, according to patient stratification on the PI3K/PTEN/Akt activation status [70]. We propose that the combination of the phosphorylation status of Akt with Tau expression may be helpful to better predict treatment efficacy.

## 5. Conclusions

**Figure 8:**
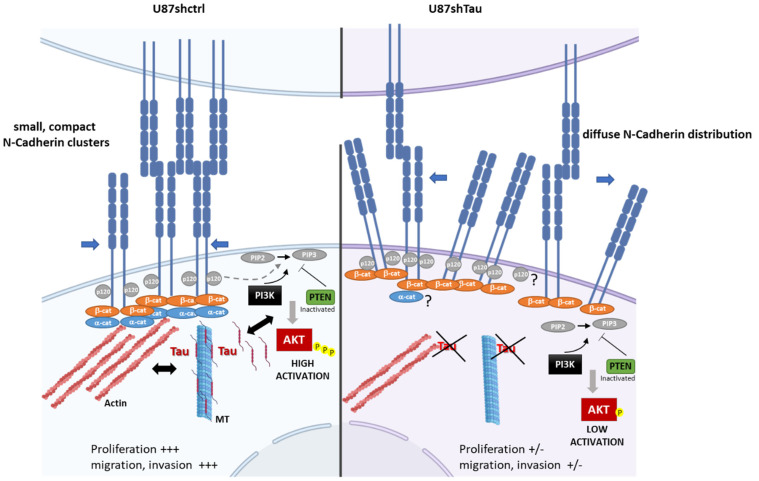
Proposed model for the role of Tau in controlling 3D N-cadherin-dependent cell cohesion and PI3K/AKT signaling axis. Left part: In the context of *PTEN*-null mutated GBM, Tau may contribute by MT-dependent function and MT-actin crosstalk to stabilize the N-cadherin-β-catenin complexes. This induces the formation of compact N-cadherin clusters reinforcing cell–cell interactions. Tau may also contribute to PI3K-AKT activation either by MT-independent interactions (direct?) or through increased N-cadherin-β-catenin signaling (indirect?). This will promote cell proliferation and invasion. Right part: In Tau-depleted cells (U87shTau), N-cadherin-β-catenin complexes recruitment to the actin cytoskeleton is defective, and N-cadherin is not stabilized at the membrane resulting in loss of compact N-cadherin clusters organization. Mislocalized N-cadherin may sequester β-catenin, which will be responsible for the loss of cell–cell cohesion, spheroid compactness and decreased PI3K-AKT signaling activity. This will result in the inhibition of cell proliferation and migration. ABBREVIATION: β-cat: β-catenin; α-cat: α-catenin; PIP-2: phosphatidyl-inositol 4,5; PIP3: phosphatidylinositol-3,4,5-trisphosphate PTEN: Phosphatase and TENsin homolog. p120, α-cat?: remains to be studied.

Our findings, beside the well-known function of Tau in controlling MT stability and cell motility, suggest a new role for Tau in glioblastoma by controlling 3D cell organization and functions via the N-cadherin- PI3K/AKT signaling axis (see the model in Figure 8). In this way, according to the cellular context, Tau may contribute to glioblastoma progression as a differential tuning of Akt activation in PTEN mutated gliomas.

## Data Availability

Data and information are included in the article or supplementarymaterials or are available from the authors upon reasonable request.

## Supplementary Materials

The following are available online at https://www.mdpi.com/article/10.3390/cancers13225818/s1, Figure S1: Tau down-regulation does not affect cell aggregation into MCS, Figure S2: Tau down-regulation reduces MCS cell invasion, Figure S3: Tau down-regulation does not affect integrin-dependent cell–matrix adhesion, Figure S4: Tau down-regulation does not affect N-cadherin expression and localization at the cell–cell contact in 2D cell culture. Video S1: Time-lapse video microscopy of U87shctrl MCS evasion, Video S2: Time-lapse video microscopy of U87shTau MCS evasion.
