# Supplementary material for: Tau Regulates Glioblastoma Progression, 3D Cell Organization, Growth and Migration via the PI3K-AKT Axis
Source: Cancers (Basel). 2021 Nov 19;13(22):5818. doi: 10.3390/cancers13225818 (PMC8616151; doi:10.3390/cancers13225818)
Supplement: Supplementary file 1 [file cancers-13-05818-s001.zip › article Tau cancers Supll figures v3.pdf]

Supplementary material

Tau regulates glioblastoma progression, *3D* cell organization, growth and migration via the PI3K-AKT axis.

Alessandra Pagano [1], Gilles Breuzard [1], Fabrice Parat[1], Aurélie Tchogandjian[2], Dominique Figarella-Branger[2], Tiphany Coralie De Bessa[3], Françoise Garrouste [1], Alexis Douence[1], Pascale Barbier[1] and Hervé Kovacic [1]

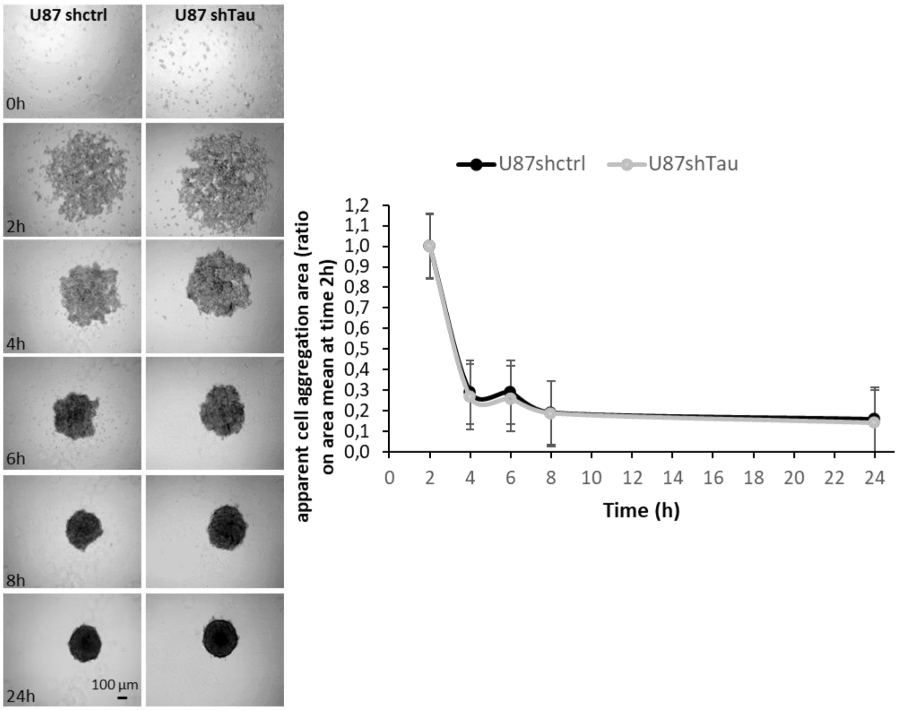

Figure S1. Tau down-regulation does not affect cell aggregation into MCS. Time course of cell aggregation into spheroid. Left: representative images of cell in methylcellulose at different time points. Right: quantification of the area occupied by aggregating cells. Area values are expressed as ratio on area mean at time 2h (mean ± SEM, N=8 spheroids).

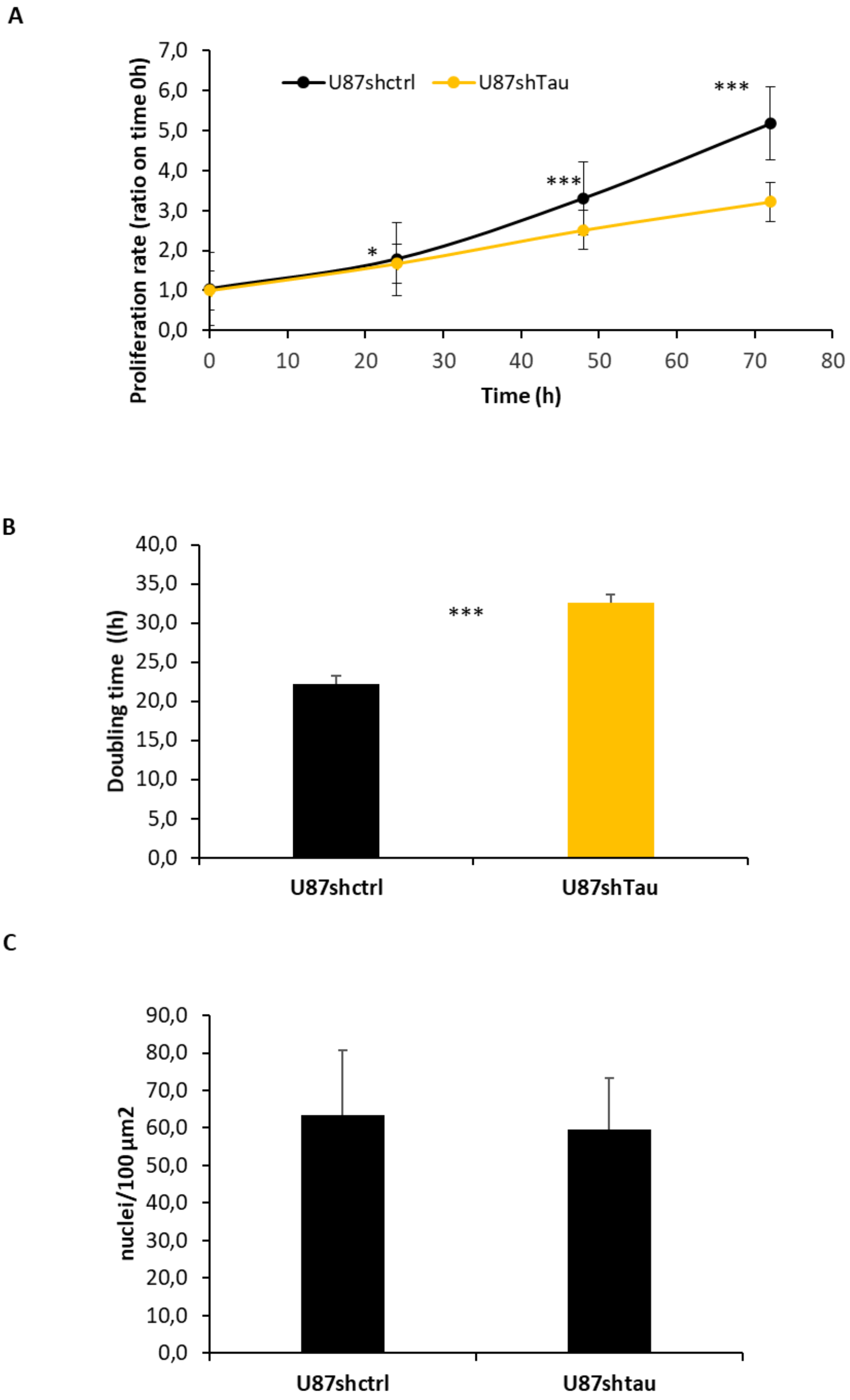

Supplementary Figure S2: Tau down-regulation increases cell proliferation rate and doubling time but not modifies cell area. A. Measure of proliferation rate: viability assay (MTT) was performed on U87shctrl and U87shTau adherent cells (2D) at time 0-24-48-72h after plating. Data are expressed as ratio of OD_{600} at each time point on OD_{600} at time 0h (mean ± SEM, N=8 for each condition; * p<0.05 24h U87shctrl vs U87shTtau, p<0.001 48,72h U87shctrl vs U87shTau). **B. Doubling time** was calculated from linear regression equation of proliferation curves; data are expressed as mean ± SEM (N=8, ***p<0.001 U87shctrl vs U87shTau. **C. Measure of nuclei on MCS area sections**. MCS were MetOH fixed, OCT embedded, cryopreserved and cryosections stained with Draq5 dye for nuclei (see material and method section). The area of MCS section was measured and cell nuclei counted. Results are expressed as the number of nuclei per 100 µm of area spheroid section (mean ± SEM, N=14, p=0,58).

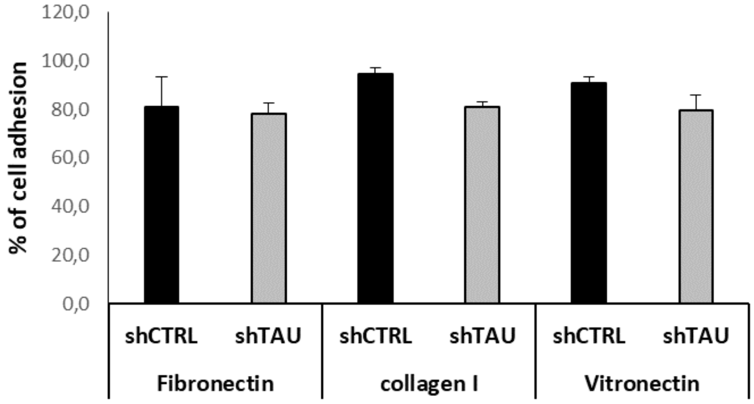

Figure S3. **Tau down-regulation does not affect cell adhesion on extracellular matrix (ECM).** Cells were plated in 96-well microtiter plates coated with 10 µg/mL fibronectin, type I collagen or vitronectin and allowed to adhere for 30 minutes at 37°C. After washing, adherent cells were stained with crystalviolet, solubilized by SDS and absorbance was measured at 600 nm. Results are expressed as % of total cell adhesion, mean ± SEM.

.

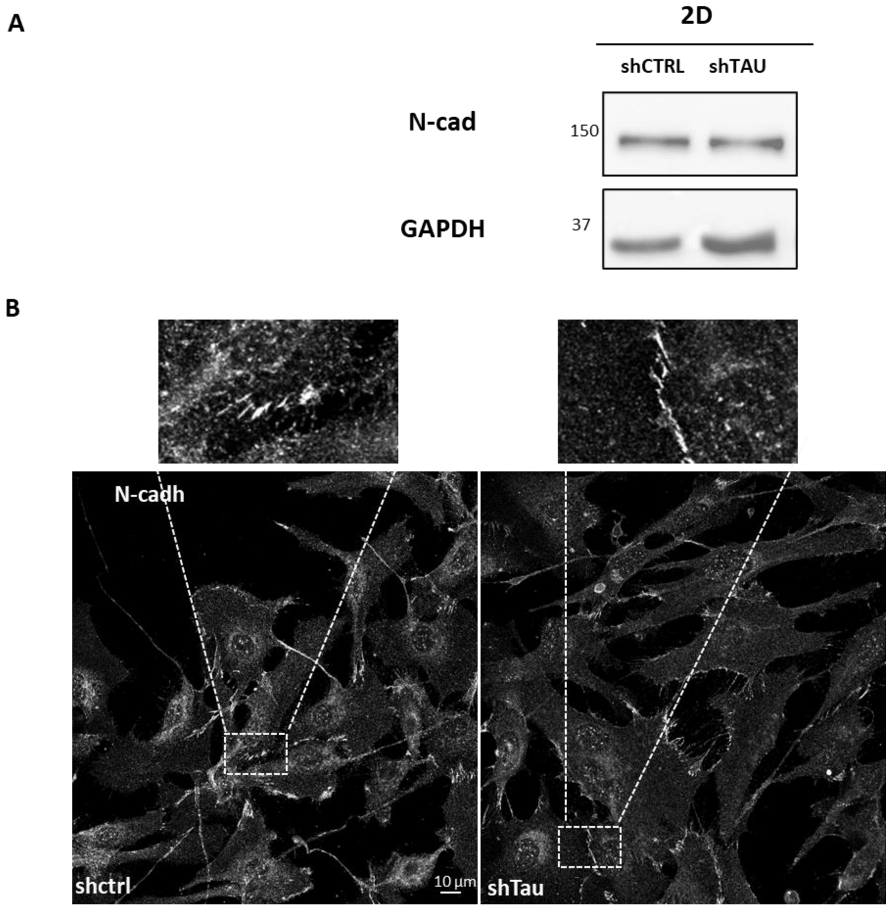

Figure S4. Tau down-regulation does not affect N- cadherin total protein expression and localization at the cell –cell contact in 2D cell culture. (A) Western blot analysis of N-cadherin expression in total cell protein lysates (30 µg). GAPDH was used as loading control. (**B**) Confocal microscopy images of N-cadherin of MetOH-fixed cells (obj 63X). The upper images show a high magnification of the boxed areas below.

Legend for movies

Video 1. Time course of U87shtcrl MCS evasion. Spheroids were settled on fibronectin (10μg/ml). Images were taken every 1h for 24h. A stack of 25 images is shown.

Video 2. Time course of U87shTau MCS evasion. Spheroids were settled on fibronectin (10μg/ml). Images were taken every 1h for 24h. A stack of 25 images is shown.
