# Supplementary material for: Tau Regulates Glioblastoma Progression, 3D Cell Organization, Growth and Migration via the PI3K-AKT Axis
Source: Cancers (Basel). 2021 Nov 19;13(22):5818. doi: 10.3390/cancers13225818 (PMC8616151; doi:10.3390/cancers13225818)
Supplement: Supplementary file 1 [file cancers-13-05818-s001.zip › article Tau cancers Suppl. methodsV3.pdf]

Supplementary material

Tau regulates tumor progression, three-D cellular spheroid organization, growth and evasion in glioblastoma via the N-cadherin -PI3K-AKT axis.

Alessandra Pagano [1], Gilles Breuzard [1], Fabrice Parat[1], Aurélie Tchogandjian[2], Dominique Figarella-Branger[2], Tiphany Coralie De Bessa[3], Françoise Garrouste [1], Alexis Douence[1], Pascale Barbier[1] and Hervé Kovacic [1]

Supplementary methods

MCS aggregation assay. For time course analysis of MCS formation, cells (**5000** cells per well) were let to aggregate for 24hs to form MCS in 96-well suspension culture U bottom plates, as described in material and method sections. Cell aggregation in methylcellulose analysed by a wide-field inverted microscope, equipped with an air objectif 4x and connected to a CCD camera, as described in material and method sections. One image per MCS was taken at 0-2-4-6-8 and 24 hours. The area occupied by aggregating cells was measured by Image J software and expressed as the ratio between the area at each time point on area mean at time 2h.

Viability assay (MTT). U87shctrl and U87shTau cells were plated in 96p well plates (5000 cells/well). At time 0-24-48-72h after plating, Thiazolyl Blue Tetrazolium Bromide was added (MTT reagent, Sigma -Aldrich 0.5 mg/ml final concentration) and incubated for 2hr. Reagent was removed, and formazan precipitates were dissolved with DMSO. OD_{600} was measured by a spectrophotometer.

Cell adhesion assay to ECM. Cell adhesion assay was performed as previously described (Delamarre *et al*, 2009) briefly, flat bottom 96-well microtiter plates (maxisorp, Nunc) were coated with one of the following purified extracellular matrix (ECM): fibronectin (10 µg/ml), vitronectin (10 µg/ml), type I collagen (10 µg/ml) and were blocked with BSA. U87shctrl and U87shTau cells were harvested in single cell suspension, suspended in EMEM containing 0.2% BSA (adhesion buffer) and allowed to adhere 30 minutes to the substrate at 37°C. Unattached cells were removed by gently washing three times with adhesion buffer. Residual attached cells were fixed by 1% glutaraldehyde, stained by 0.1% crystal violet and lysed with 1% SDS. Absorbance was then measured at 600 nm by a microplate reader. Results were expressed as % of total unwashed cell adhesion (mean ± SEM n=3, more than 3 experiments).

Immunofluorescence of 2D-cell culture- Immunostaining of N-cadherin was done according protocols previously described (Breuzard *et al*, 2019). Antibodies used were the same as described in material and method section.
