# Supplementary material for: Tau Regulates Glioblastoma Progression, 3D Cell Organization, Growth and Migration via the PI3K-AKT Axis
Source: Cancers (Basel). 2021 Nov 19;13(22):5818. doi: 10.3390/cancers13225818 (PMC8616151; doi:10.3390/cancers13225818)

RAW western blot

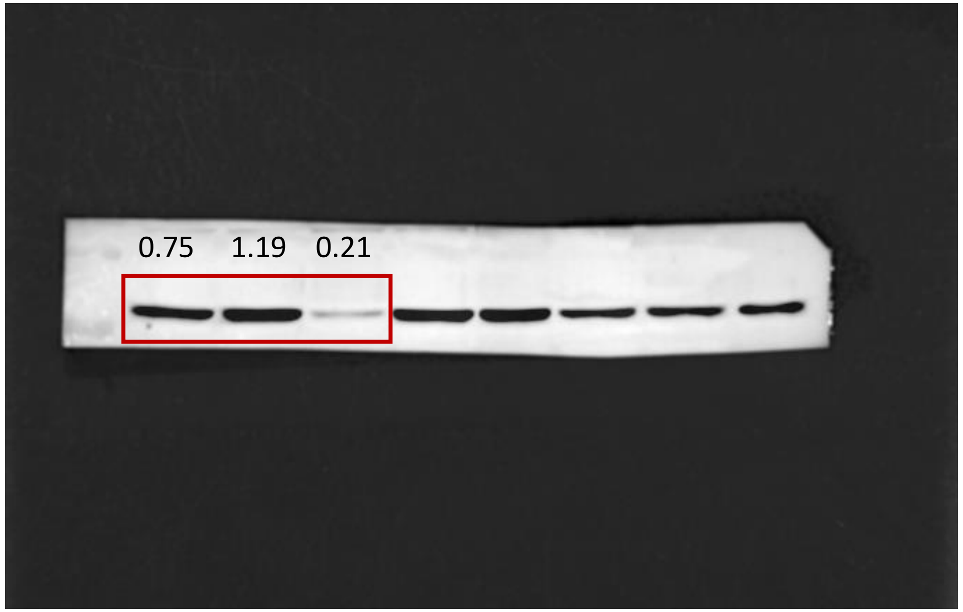

TAU

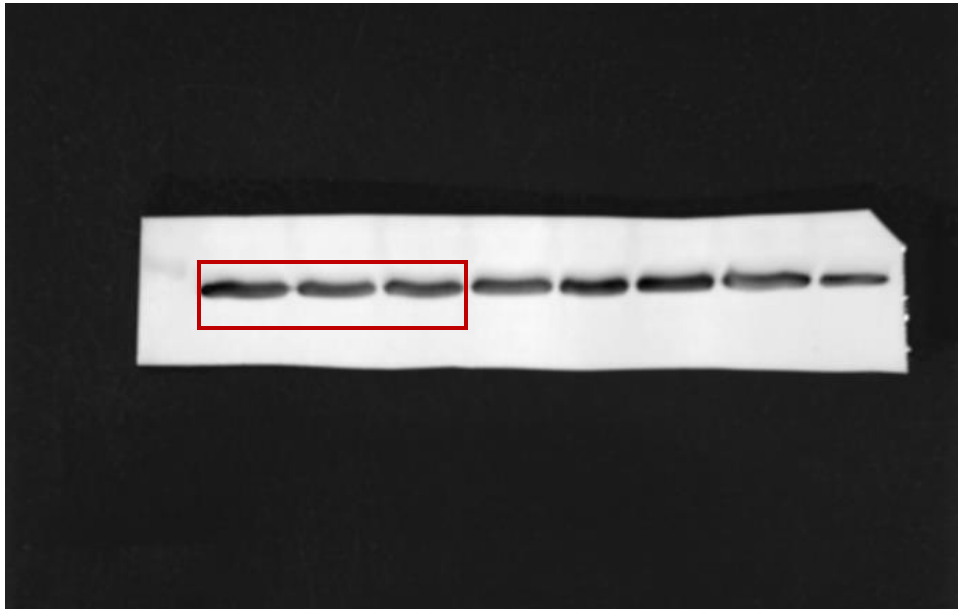

GAPDH

Figure 1A

Integrated density values (ratio on GAPDH values)

Figure 4A

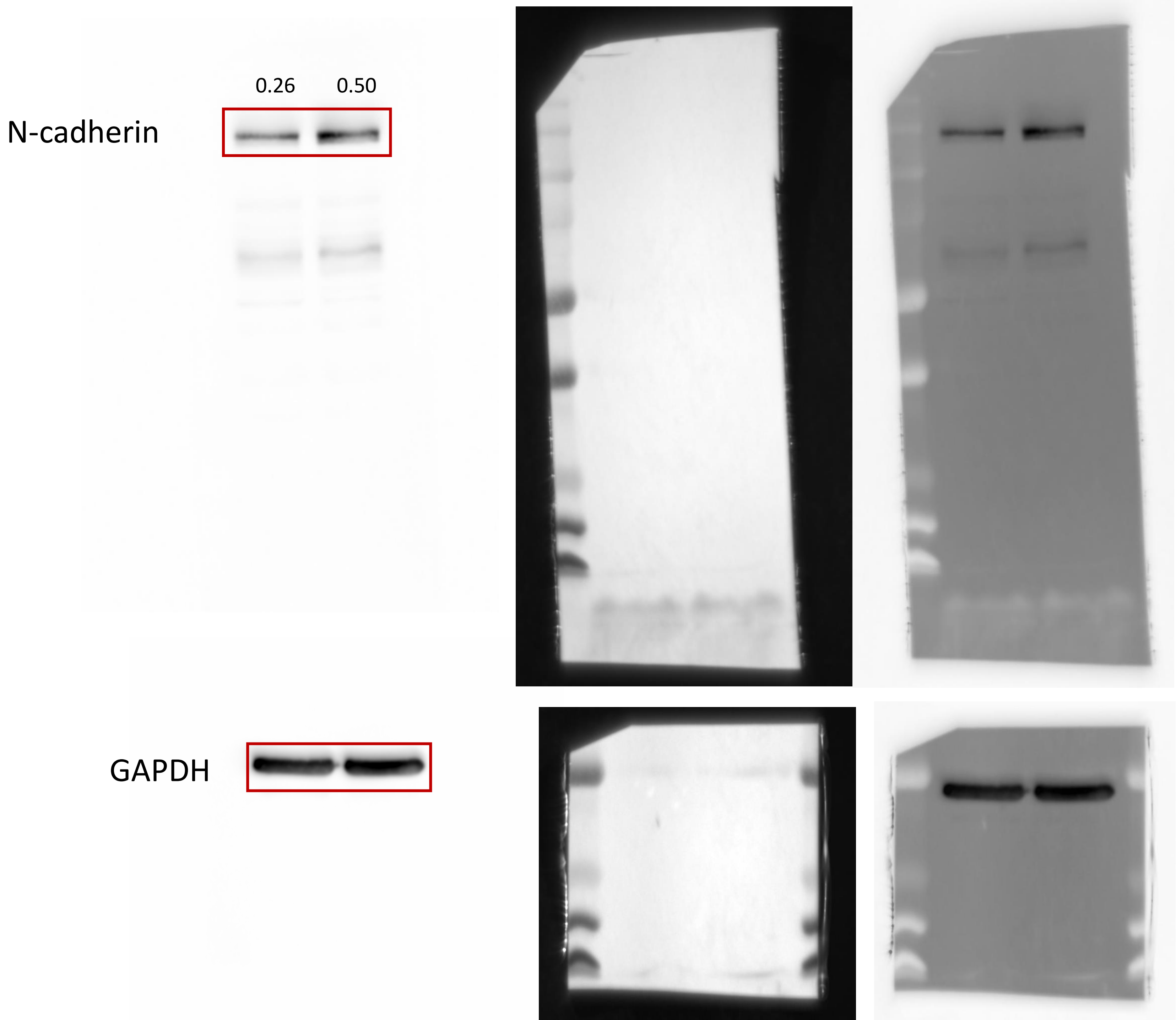

Figure 4A

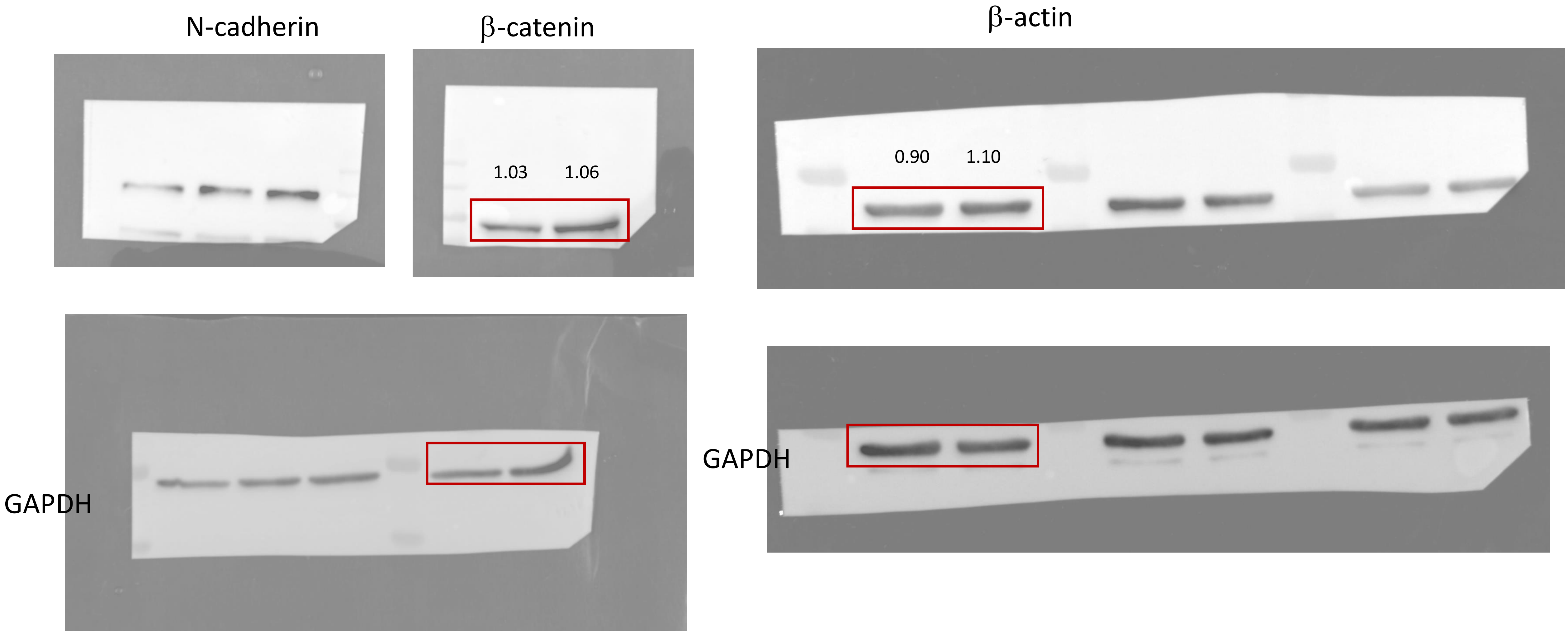

Figure 6B

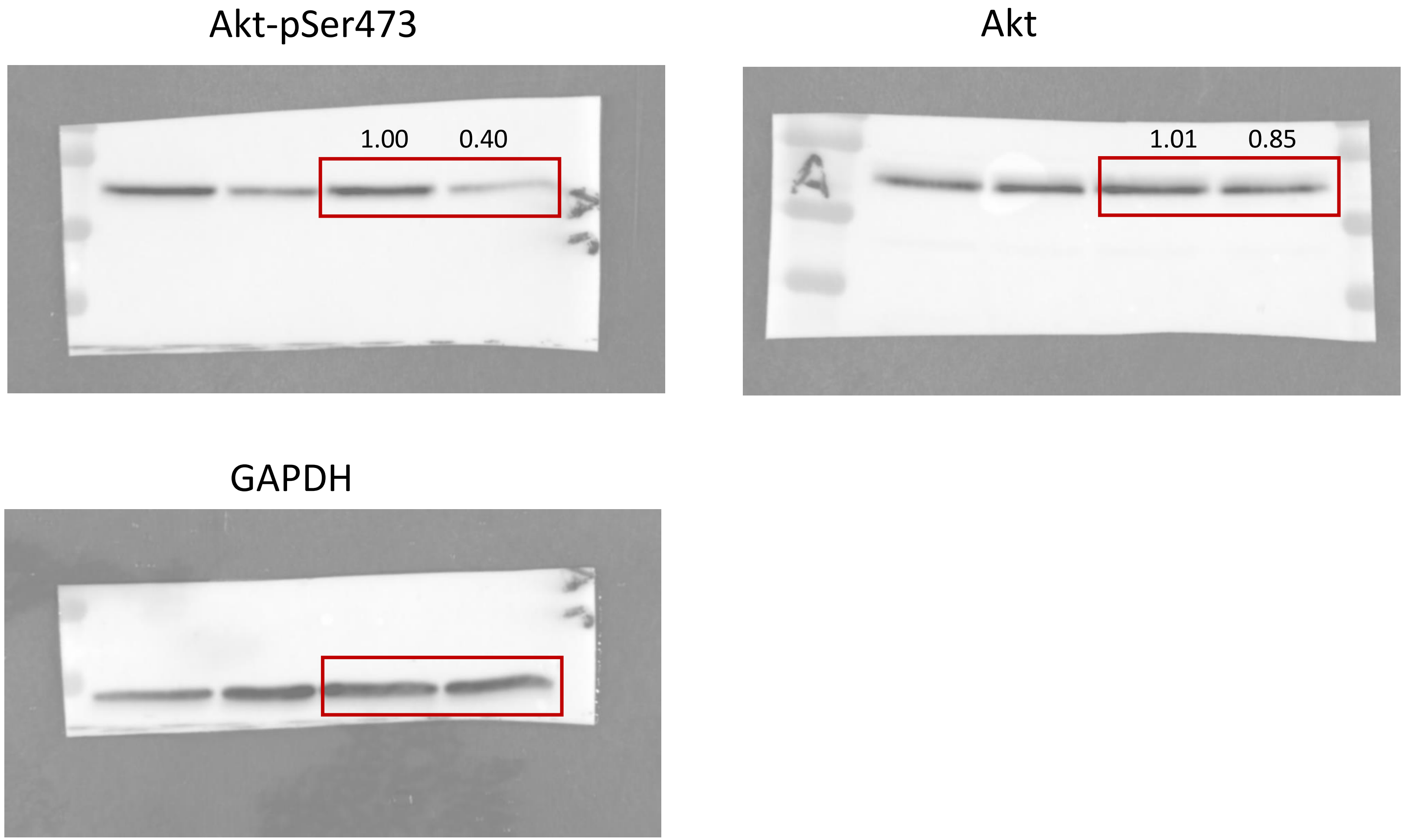

Figure 6B

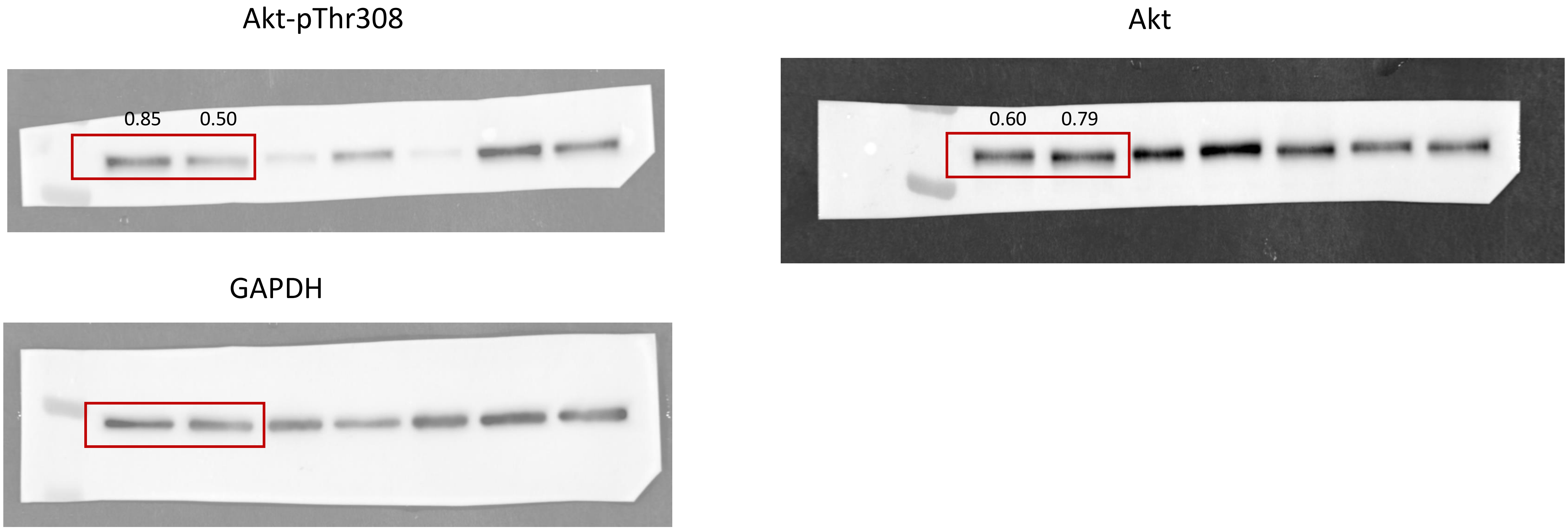

Figure S4

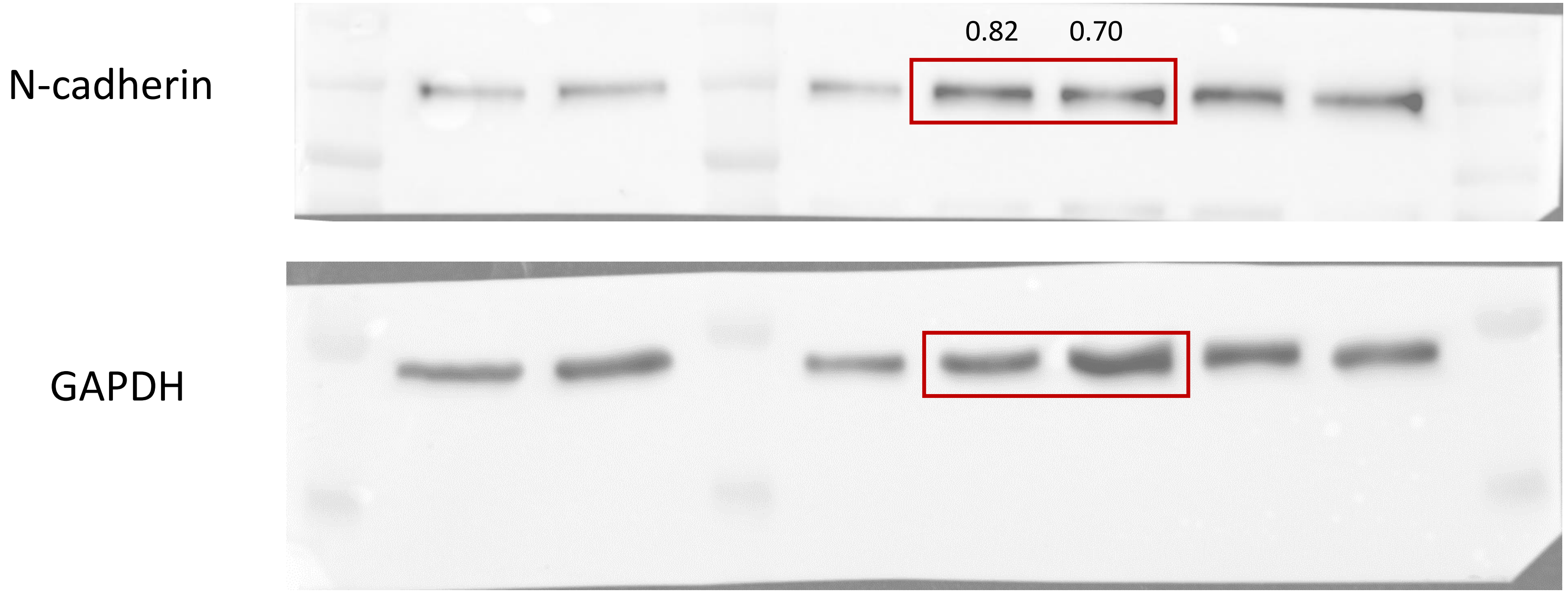

Supplement: Supplementary file 1 [file cancers-13-05818-s001.zip › original western blots+densitometric values 16.11.21.pdf]
